# Is Childhood Trauma Related to Stress-related Exhaustion in Adulthood? A Pilot Study Based on a Consecutive Clinical Sample of Patients with Stress-related Exhaustion

**DOI:** 10.1007/s40653-026-00891-6

**Published:** 2026-04-30

**Authors:** Johan Möller, Susanne Ellbin, Anna Ehnvall, Ann-Sophie Lindqvist Bagge, Ingibjörg H. Jonsdottir

**Affiliations:** 1 The Institute of Stress Medicine, Gothenburg, Region Västra Götaland Sweden; 2 Section of Psychiatry, Institute of Clinical Neuroscience, Gothenburg University, Gothenburg, Sweden; 3 Psychiatric Out-patient Clinic, Region Halland, Varberg, Sweden; 4 Department of Psychology, Gothenburg University, Gothenburg, Sweden; 5 School of Public Health and Community Medicine, Institute of Medicine, Sahlgrenska Academy at the University of Gothenburg, Gothenburg, Sweden

**Keywords:** Burnout, Exhaustion, Childhood trauma, Emotional abuse, Emotional neglect, Stress-related exhaustion

## Abstract

The present study aims to explore whether self-rated childhood trauma is related to stress-related exhaustion in adulthood. A naturalistic study based on a consecutive clinical sample of patients with stress-related exhaustion (*n* = 38) and non-clinical controls (*n* = 33). Early adversity was measured by using the Childhood Trauma Questionnaire (CTQ). The degree of stress-related exhaustion was assessed by the Shirom-Melamed Burnout Questionnaire (SMBQ). Patients scored significantly higher on the emotional neglect CTQ-subscale (mean 12.2 (SD 5.0) than controls (mean 9.1 (SD 3.9) (*p =* 0.008) whereas other subscales did not differ. The total mean score for CTQ did not differ between patients and controls. A higher percentage of patients scored above the cut-off for the subscales emotional neglect (73% compared to 36%) (*p =* 0.004) and emotional abuse (56% compared to 26%) (*p =* 0.019) compared to the controls. CTQ subscales emotional neglect and emotional abuse were also significantly related to SMBQ (*r* = 0.350, *p =* 0.05) whereas other CTQ subscales were not related to levels of exhaustion in the patient group. Emotional neglect in childhood is more frequently reported by patients with ED than by individuals in the non-clinical population. These findings suggest that, when clinically relevant, a history of emotional neglect should be addressed in the treatment of patients with ED, particularly among those with long-standing symptoms. However, the present study included a small sample size, and the findings require confirmation in larger, adequately powered studies.

## Introduction

Early life adversity has previously been associated with an increased risk of psychiatric disorders later in life. However, it remains unclear whether this association also applies to patients with clinical burnout or stress-related exhaustion. Thus, the relationship between early life adversity and an increased risk of mental disorders, such as depression, anxiety, and substance abuse, has previously been reported in several studies (Felitti et al., 1998; Humphreys et al., 2020; Norman et al., 2012; Salum et al., 2010). A meta-analysis showed a particularly strong association between emotional abuse during childhood and depression later in life (Humphreys et al., 2020). Previous studies have also observed that adverse childhood experiences (ACE) increase vulnerability to psychiatric disorders following a stressful life event (Hammen et al., 2000; Harkness et al., 2006; McLaughlin et al., 2010). Thus, it is fairly well known that early life adversity is related to many different psychiatric disorders. However, it is of interest to see whether a similar relationship is observed among individuals with stress-related mental health problems including burnout/exhaustion. Stress-related mental health disorders are considered a global problem with a significant economic burden (Hassard et al., 2018). A study from the Netherlands showed that sick leave due to stress-related illness places a heavy burden on employers and thus high costs for society (Wolvetang et al., 2022). For example, in the United States, reduced productivity due to stress-related absenteeism of physician’s alone costs USD 4.6 billion per year (Han et al., 2019). Thus, increasing the knowledge of factor of contributing factors to stress-related mental health is urgent.

A variety of partly overlapping concepts are being used in the literature to define burnout/exhaustion. The burnout concept has been differently defined depending on the theoretical background (Shirom & Melamed, 2006). The World Health Organization defines burnout as a syndrome resulting from chronic workplace stress that has been unsuccessfully managed (WHO, 2019). An international consortium of researchers from more than 35 countries applies a slightly different definition, defining burnout as a work-related state of exhaustion adding that problems outside the work domain and/or personal vulnerability may facilitate the development of burnout (Desart & De Witte, 2019). It also varies between countries which definition is used in clinical practice and concerns have been raised that burnout as a concept is not usable as a diagnostic tool in clinical practice (Kleijweg et al., 2013). In 2003, the clinical criteria for the diagnosis exhaustion disorder (ED) were presented in Sweden and from 2005 the code F43.8 A in ICD-10 has been recommended to be used to classify ED. The condition is attributed to long-term stress exposure and lack of recuperation resembling clinical burnout with symptoms of severe mental and physical fatigue, cognitive impairment and sleep disturbances. Studies have shown that exposure is often related to both work and non-work factors, with work-related factors playing a dominant role for most patients (Hasselberg et al., 2014).

Several studies have explored the relationship between burnout and adverse childhood experiences. The populations included in these studies are various working populations with none of the studies including clinical populations with burnout/exhaustion. A large study including physicians reported that individuals reporting adverse childhood experiences were more likely to report burnout and those with an ACE score of 4 or more had more than two and a half times the risk of reporting burnout (Yellowlees et al., 2021). Similar results were seen in a large population of healthcare workers, showing a relationship between adverse childhood experiences and emotional exhaustion (Clemens et al., 2021) and among nursing students (McKee-Lopez et al., 2019). and a study by Mather et al. (2014) observed that a history of traumatic life events was associated with later burnout symptoms, with a cumulative effect with an increasing number of events (Mather et al., 2014).

The lack of studies on clinical populations raises the need to add to the literature knowledge regarding plausible childhood trauma in a clinical patient population seeking care for stress-related problems. The cause of illness is linked to work-related and/or private-related stress exposure. However, individual factors—such as personality traits and early life experience may further complicate the situation and should be considered when assessing and treating these patients.

The general aim of this study was to explore whether childhood trauma was related to stress-related exhaustion in adulthood. The specific research question was to study whether patients diagnosed with stress-related exhaustion in adulthood differed in the prevalence of self-reported childhood trauma compared to non-clinical controls. We hypothesize that patients with stress-related exhaustion will report some aspects of early adversity that will differ from healthy controls.

## Methods

### Participants and procedure

#### Participant – patients

Patients were recruited from a specialist outpatient clinic for patients with stress-related exhaustion in the Western region of Sweden. Patients were referred to the clinic from primary care units or occupational health care centers between November 2010 and October 2011. To ensure a relatively homogenous study population, all patients that were included in this study fulfilled the diagnostic criteria for Exhaustion Disorder (Table 1), diagnostic criteria used by clinicians in Sweden to define patients with clinical burnout (Grossi et al., 2015). Patients were recruited consecutively during ordinary follow-up visits to the clinic, and 60 patients were asked to participate in the study. After receiving the written information and reviewing the questionnaires, one patient declined participation verbally due to clinical issues. The inclusion occurred three to six months after the start of a multimodal treatment program at the clinic. The treatment procedure has previously been described in detail (Glise et al., 2012, 2014). Briefly, after a thorough medical assessment, all patients received individually tailored treatment over approximately 18 months. They met with a physician every fourth to six weeks, with intervals increasing over time. Psychological consultations and psychotherapy were provided when needed. All patients received advice on lifestyle factors—such as physical activity and sleep. Psychoeducation and/or stress reduction groups were offered early in treatment, and employers, colleagues, and relatives were invited to a brief lecture on stress related disorders. Antidepressants were prescribed when clinically indicated.

**Table 1:** Diagnostic criteria for Exhaustion Disorder according to the Swedish National Board of Health and Welfare

A	Physical and mental symptoms of exhaustion with minimum two weeks duration. The symptoms have developed in response to one or more identifiable stressors which have been present for at least 6 months.
B	Markedly reduced mental energy, which is manifested by reduced initiative, lack of endurance, or increase of time needed for recovery after mental efforts.
C	At least four of the following symptoms have been present most of the day, nearly every day, during the same 2-week period:
*1*	*Persistent complaints of impaired memory.*
*2*	*Markedly reduced capacity to tolerate demands or to work under time pressure.*
*3*	*Emotional instability or irritability.*
*4*	*Insomnia or hypersomnia.*
*5*	*Persistent complaints of physical weakness or fatigue.*
*6*	*Physical symptoms such as muscular pain*,* chest pain*,* palpitations*,* gastrointestinal problems*,* vertigo or increased sensitivity to sounds.*
D	The symptoms cause clinically significant distress or impairment in social, occupational or other important areas of functioning.
E	The symptoms are not due to the direct physiological effects of a substance (e.g. a drug of abuse, a medication) or a general medical condition (e.g. hypothyroidism, diabetes, infectious disease).
F	If criteria for major depressive disorder, dysthymic disorder or generalized anxiety disorder are met, exhaustion disorder is set a co-morbid condition.

The patients were outpatients at the time of the study, and none had received inpatient care for their illness. The criteria for participation in the study were the same as the clinic’s initial patient acceptance criteria: the patient had to meet the ED diagnostic criteria and have been on sick leave for no more than six months. Co-morbidity of depression and/or anxiety was allowed and screened for by using the one-page Primary Care Evaluation of Mental Disorders questionnaire (Spitzer et al., 1994). This was followed by a physician using a structured interview form conforming to the DSM IV criteria for diagnostic assessment of mood and anxiety disorders (*Diagnostic and Statistical Manual of Mental Health Disorders* 1994). All patients fulfilled the criteria for ED, whereas comorbid depression and anxiety were observed in twenty-six patients (68%). A further four patients fulfilled the criteria for co-morbid depression only and another four fulfilled the criteria for co-morbid anxiety only.

#### Exclusion Criteria

Patients with generalized pain, fibromyalgia, chronic fatigue syndrome/myalgic encephalomyelitis, thyroid disease, vitamin B-12 deficiency, alcohol/drug addiction, other psychiatric illnesses other than depression and anxiety, and other somatic diseases that could explain fatigue were not accepted as patients at the clinic. Nineteen patients did not return the questionnaire, and two patients were excluded because they did not fulfill the diagnostic criteria for ED.

#### Participant – controls

Non-clinical controls were recruited from a technology company in the same city in Sweden as the clinic. The inclusion criteria for controls were being mentally healthy, i.e. no apparent psychiatric disorder, and the exclusion criteria for controls comprised self-reported exhaustion measured using a four-item self-rating scale strictly based on the diagnostic criteria for ED. This self-reported scale has been shown to capture mental health problems which have been shown to correspond well to established scales for mental health, indicating sufficient construct validity as well as predictive validity for future sick-leave and poor work ability (Glise et al., 2010). The non-clinical controls were also asked to complete the Comprehensive Psychopathological Rating Scale (CPRS) to ensure that the most common psychopathology was not present (Asberg et al., 1978). Forty individuals received an envelope containing the questionnaires, and four did not return the questionnaires. Three controls were excluded because they reported exhaustion according to the self-rated ED scale.

### Measures

#### Childhood Trauma

The statistical validity and reliability of the self-rated Childhood Trauma Questionnaire (CTQ) have previously been shown (Bernstein et al., 1994) and the questionnaire has subsequently been revised as a short form and translated into Swedish (Gerdner & Allgulander, 2009). The Swedish version of CTQ-SF includes 28 items measuring: ‘Emotional abuse’, ‘Physical abuse’, ‘Sexual abuse’, ‘Emotional neglect’, and ‘Physical neglect’, each of which includes five items. The subscale, ‘Minimization/denial’, is also included in the CTQ and contains three items. It is used to detect underreporting of maltreatment or denial of conceivable childhood trauma, and it is highly recommended that the outcome from this subscale is considered when assessing ACE, using CTQ (Church et al., 2017; MacDonald et al., 2016). Individuals scoring five for all three items on this subscale were excluded from the CTQ analysis (Bernstein & Fink, 1998). The CTQ was developed as a five-point Likert scale, ranging from one (never true) to five (very often true). The original classification of the cut-off for ‘abuse/neglect’ or ‘no abuse/neglect’ was based on calculations of percentiles of young addicts (Bernstein & Fink, 1998) with higher scores being interpreted as more abuse/neglect. Cronbach’s alphas (*α*) for the subscales in this study are as follows: ‘Emotional abuse’ (*α* = 0.860), ‘Physical abuse’ (*α* = 0.896), ‘Sexual abuse’ (*α* = 0.806), ‘Emotional neglect’ (*α* = 0.911), ‘Physical neglect’ (*α* = 0.593) and ‘Minimization/denial’ (*α* = 0.734).

#### Symptoms of Burnout

The Shirom-Melamed Burnout Questionnaire (SMBQ) has been found useful to measure symptoms related to exhaustion among patients with stress-related exhaustion (Glise et al., 2012; Lundgren-Nilsson et al., 2012). The global SMBQ score correlates to a high degree with the Maslach Burnout Inventory (MBI) and has been shown to possess good construct validity and reliability (Grossi et al., 2003). Originally, the instrument contained 22 items and four subscales: physical fatigue (eight items), cognitive weariness (six items), tension (four items), and listlessness (four items). In this study, a revised 18-item version (tension excluded) was used which has been proven to have good construct validity (Lundgren-Nilsson et al., 2012). Each item is answered on a scale with seven ordered categories ranging from ‘rarely’ to ‘almost always’. Instead of the mean score of the 18 items, a recommended Rasch-transformed score was calculated (Lundgren-Nilsson et al., 2012). This score ranges from 18 to 126, with higher values indicating a high degree of burnout, and a value of 79 cut-off for clinical burnout. Cronbach’s alpha for the total score of SMBQ was found to be 0.93.

#### Anxiety and Depression

*Comprehensive Psychopathological Self- rating Scale for Affective Syndromes (CPRS-S-A)* is a self-rating scale for affective syndromes, constructed from the original CPRS scale, which is well validated and covers a wide range of mental symptoms (Asberg et al., 1978). The self-rating scale consists of 19 items measuring depression, anxiety, and obsessional symptoms, re-phrased from the original instrument. It has been found to have a high degree of concordance with the original instrument for most subscales and is considered useful in a quantitative rating of symptoms in ambulatory patients, both in research and clinical setting (Svanborg & Asberg, 1994). Cronbach´s alpha for CPRS was 0.92. The CPRS manual does not specify a cut-off score for determining the likelihood of a clinical condition. When applied appropriately, the scale should be regarded as a supplementary tool for clinical assessment rather than as a standalone diagnostic instrument. In the present study, the scale was used to provide an approximate indication of differences between the patient group and the normative population.

### Statistical Analysis

A Student t-test was used to compare mean total scores between patients and controls on both scales. The chi-square test was used to determine whether the number of patients and controls who responded was high/low across different CTQ subscales. Pearson correlation test was used to analyze correlation. One patient had missing data on the emotional neglect subscale and one patient had missing data on the emotional abuse subscale. No imputation of data was performed.

### Ethical Consideration

The study was approved by the regional ethical review board in Gothenburg, Sweden (#398–06). All participants gave their written informed consent before entering the study.

## Results

The final number included was 38 patients (82% women), aged 21 to 65 and the final sample of controls totaled 33 participants (70% women) aged from 21 to 65 years (Table 2). Consecutive inclusion from a patient cohort was applied in this study and the patients included (*N* = 38) did not differ from the general patient cohort that was registered as a patient at the clinic when the study was conducted (*N* = 92) regarding age, percentage of females/male or severity of exhaustion (data not shown). The patient group included in the study reported on average 16 years of education (SD 2.8). The patient group differed significantly from the control group on all CPRS measures, indicating a higher overall level of psychiatric symptoms in the patient group. (See Table 2).

**Table 2:** Demographics and mental health scores for patients and controls

		Patients (*n* = 38)	Controls (*n* = 33 )	*p*-value
Age, *n* (%)^3^				0.064
	21–30 years	4 (11)	1 (3)	
	31–40 years	13 (34)	4 (12)	
	41–50 years	11 (29)	14 (42)	
	51–65 years	10 (26)	14 (42)	
Sex, n (%)^3^				0.242
	Female	31 (82)	23 (70)	
	Male	7 (18)	10 (30)	
CPRS^1^ score, mean (*SD*)^4^				
- Depression		7.9 (3.1)	3.3 (2.9)	< 0.001
- Anxiety		9.3 (3.4)	5.0 (3.6)	< 0.001
- Total score		18.1 (7.0)	8.2 (6.5)	< 0.001
Clinical Burnout (> 4.4 for SMBQ^2^)		92%	NM^5^	

^1^CPRS = Comprehensive Psychopathological Rating Scale

^2^SMBQ = Shirom-Melamed Burnout Questionnaire

^3^Age and sex were analyzed using the chi-square test

^4^The student’s t-test was used to analyze CPRS scores

^5^NM = Not measured

### Childhood Trauma in Patients with Exhaustion Disorder

In total, nine participants (four patients and five controls) were excluded due to minimization/denial. The total mean score for the CTQ did not differ between patients (*M* = 39.7, *SD =* 10.0) and controls (*M* = 34.7, *SD =* 13.5) (*p =* 0.106). The patients scored significantly higher on the emotional neglect CTQ-subscale (mean 12.2 (SD 5.0) compared to mean 9.1 (SD 3.9) (*p*=0.008) (mean difference 3.1 (95% CI 0.8–5.4)). The mean scores for the other CTQ subscales did not differ between patients and controls. A higher percentage of the patients scored above the cut-off for the subscales Emotional neglect (*p*=0.004) and Emotional abuse (*p*=0.019) compared to the controls. (See Table 3).

**Table 3:** Mean and percentage scores above the cut-off on different subscales from the Childhood Trauma Questionnaire (CTQ)

CTQ^1^ Subscales	Mean score (SD)^2^			Number (%)above cut off^3^		
	Patients (*n* = 34)	Controls (*n* = 28)	*p* value	Patients (*n* = 34)	Controls (*n* = 28)	*p* value
Emotional abuse	9.2 (3.9)	7.6 (4.3)	0.123	19 (56)	7 (26)	0.019
Physical abuse	5.9 (1.8)	6.3 (3.9)	0.645	5 (15)	3 (11)	0.641
Sexual abuse	5.5 (1.5)	5.3 (1.0)	0.572	5 (15)	2 (7)	0.349
Emotional neglect	12.2 (5.0)	9.1 (3.9)	0.008	24 (73)	10 (36)	0.004
Physical neglect	7.2 (2.3)	6.6 (3.1)	0.406	13 (38)	5 (18)	0.079

^1^CTQ = Childhood trauma Questionnaire

^2^The student’s t-test was used to analyze the differences in means

^3^Chi-square test was used to analyze the differences between cut-offs

### Childhood Trauma and Level of Exhaustion among the Patients

Correlation analysis showed that the level of exhaustion among the patients, measured using the SMBQ, was significantly correlated to the CTQ total score (*n* = 32, *r* = 0.392, *p* = 0.027) and emotional neglect was also related significantly to exhaustion (*r* = 0.352, *p* = 0.048) whereas other subscales were not related to levels of exhaustion in the patient group (data not shown).

### Childhood Trauma and Level of Anxiety and Depression

CTQ scores, neither total scores nor scores for respective subscales, were significantly related to symptoms of depression or anxiety among the patients. Thus, for the total CTQ, the correlation analysis showed that CTQ score was not significantly related to either depression (*r* = 0.253, *p* = 0.0.177) or anxiety (*r* = 0.136, *p* = 0.474). The results from the subscales did not show significant correlation with either depression or anxiety. This was also the case for emotional neglect (depression *r* = 0.296, *p* = 0.113 and anxiety (*r* = 0.179, *p* = 0.344).

## Discussion

The present results showed that patients seeking care for stress-related exhaustion, compared to non-clinical controls, more often reported emotional neglect in childhood and were more likely to score above the clinical cut-off for self-reported emotional neglect and emotional abuse. The results further showed that the level of exhaustion reported by the patients correlated significantly with cumulative childhood trauma as well as emotional neglect and emotional abuse. However, no relation between stress-related exhaustion in adulthood and self-reported physical abuse, physical neglect, and sexual abuse in childhood was seen. Thus, the previous observed relationship between some aspects of early life adversity and psychiatric problems was also observed in a clinical patient population with stress-related exhaustion.

Interestingly, in this clinical population of patients with stress-related exhaustion, the relationship between early adversity and exhaustion was restricted to emotional neglect and some extent emotional abuse. In previous studies on working populations with burnout, overall ACE was measured, and it was thus not possible to confirm if the childhood trauma reported in these previous studies was restricted to emotional aspects as seen in this current study (Clemens et al., 2021; Mather et al., 2014; McKee-Lopez et al., 2019; Yellowlees et al., 2021). Gerder and Allgulander showed that patients with an anxiety disorder or patients with alcohol abuse, score higher on the emotional neglect CTQ-subscale, but also higher on all other CTQ subscales compared to non-clinical controls, which differs from the results in this present study (Gerdner & Allgulander, 2009). Our result indicating a relationship between childhood emotional neglect in childhood and adult exhaustion was supported by the observed correlation between exhaustion and CTQ scores.

Childhood emotional neglect, together with childhood emotional abuse, represents the least studied forms of childhood maltreatment, according to a recent meta-analysis (Xiao et al., 2023), possibly attributable to challenges related to both the conceptual definition as well as the detection of emotional forms of maltreatment (Simon et al., 2024; Xiao et al., 2023).To the best of our knowledge, there are no previous studies investigating the relationship between childhood emotional neglect and stress-related exhaustion in adulthood. In line with our findings are the results from the meta-analysis by Xiao and coworkers, showing that individuals in clinical populations are generally more likely to have experienced emotional abuse and emotional neglect during childhood compared with non-clinical populations (Xiao et al., 2023). Furthermore, research from a related field, examining adolescents attending high school and exhibiting symptoms of academic burnout—characterized by emotional exhaustion, reduced motivation, and diminished enthusiasm for schoolwork—demonstrated that parental emotional neglect was associated with academic burnout among their children (Xu et al., 2025).

The results from this study should be generalized with great care, as it includes a high percentage of non-responders among patients approached to participate. It can be speculated that some of the non-responders negated participation due to unpleasant memories from this period of life and this would mean that the occurrence of adverse childhood trauma in the patient sample is underestimated. Non-responding rates differ considerably between studies and a similar percentage of non-responders was seen in the study by Yellowlees (2021), showing a non-responding rate of 40% for physicians who chose to respond to a general questionnaire but not the adverse childhood trauma part of their study (Yellowlees et al., 2021). Thus, compared with other studies, the non-response rate in this study appeared similar. An important issue to raise regarding participation in this study is that we often face challenges recruiting patients with stress-related exhaustion to participate in research studies. These patients are normally very tired and lack the energy to complete questionnaires or perform various tests. Thus, the non-participation in this study was similar to what we have experienced in other studies and thus was not unique for this particular study (Ellbin et al., 2021).

Although the number of participants was small, some reflections on the study results including plausible mechanisms are warranted. When childhood trauma was reported by patients with stress-related exhaustion, it was mainly related to emotional neglect and in some cases emotional abuse. Emotional neglect in childhood has been shown to be related to several consequences later in life including poor coping ability, poor psychiatric resilience defined as the absence of or low psychiatric morbidity, and high psychological distress (Daníelsdóttir et al., 2022; Gruhn & Compas, 2020; Zhang et al., 2020). Emotional neglect and emotional abuse in childhood has been shown to be related to negative cognitive styles, overall emotion dysregulation as well as poor self-esteem, and a negative representation of oneself in relation to others (Briere & Jordan, 2009; Li et al., 2020). Patients with stress-related exhaustion have developed their symptoms due to long-lasting high-stress exposure that has not adequately been dealt with, including high workload, emotional demands, and/or relationship problems (Hasselberg et al., 2014). Individuals who previously experienced emotional neglect with the above-mentioned consequences are probably less prepared to cope with high emotional demands and difficult life situations. It might thus not be a surprise to observe a relatively higher number of individuals reporting adverse childhood trauma among patients with stress-related exhaustion compared to a non-clinical population. It can also be cautiously speculated that early detection of these problems is warranted as these patients risk long-term and more severe problems that will not be successfully managed if they remain. Future research should employ larger clinical cohorts to investigate whether adverse childhood experiences predict clinical outcomes in an observational framework. Adequately powered longitudinal studies are needed to establish the predictive value of early adverse experiences for long-term clinical outcomes.

Several methodological considerations including limitations should be mentioned. Firstly, it should be noted that the present study was a small cross-sectional study and no conclusion regarding causality could be drawn. The patients in the present study constituted a relatively homogeneous population characterized by stress-related exhaustion and an absence of severe psychiatric comorbidity, except for depression and anxiety. Consistent with this, participants exhibited significantly elevated levels of anxiety and depressive symptoms compared with the control group. It should be acknowledged that this psychiatric comorbidity may have influenced the findings; therefore, comparisons with other patient populations should be interpreted with caution. The level of exhaustion was significantly related to the level of emotional neglect, whereas symptoms of depression and anxiety did not seem to be related to emotional neglect in this patient population. Our research group has previously shown that symptoms of depression and anxiety decline during the first three months of treatment for stress-related exhaustion (Glise et al., 2012). Hence, we do not believe that symptoms of anxiety and depression are of major importance for the prognosis and symptom development among these patients. The low number of men included in this study prevented us from separating men and women in the analysis. Excluding participants due to possible minimization/denial did not change the analysis outcome.

Some limitations related to selection bias should be noted. Thus, most of the patients reported a higher socioeconomic status compared to the general Swedish population (Glise et al., 2012, 2014). Thus, a referral bias might have been present since the included patients might have requested specialist care from their GP to a higher extent. However, a previous study on primary care patients with the same diagnosis showed a fairly good agreement regarding the character of the patient group regarding sex, age, educational level, and burden of symptoms (Wiegner et al., 2015). Some limitations regarding the non-clinical controls should also be noted, including a lack of baseline information on socioeconomic status (i.e. income and educational level). We do acknowledge that the control group is somewhat narrow since all were recruited from the same workplace. However, we assumed that the socioeconomic status of the controls was similar to the patients, i.e. white-collar, since controls were recruited from a high-tech company that required higher education attainment for employment. Furthermore, the controls were slightly older than the patients and depending on the current age a person could modify their experience and the ability to recall childhood trauma in terms of duration, intensity, and frequency. However, the levels assessed in the present control group corresponded to previous results for other control groups (Gerdner & Allgulander, 2009). Finally, the use of self-report questionnaires to collect data regarding childhood trauma might be vulnerable to response bias, and clinical interviews would therefore be preferable. The above-described limitations, together with the high percentage of non-responders among the patients, indicate that generalizing the present result should be done with care. Thus, even though the results are in line with previous research, further well-designed studies using multiple methodologies should be performed to verify the observed results regarding the relationship between childhood trauma and stress-related exhaustion.

The main conclusion from this study was that it was more common for patients with stress-related exhaustion to report emotional neglect compared to the non-clinical population. The results were in line with previous research studying similar groups such as individuals with high burnout. This is a small study, and the results need to be confirmed in larger studies, but we can cautiously conclude that treatment of patients with exhaustion should not only focus on contemporaneous stress events but also on possible childhood maltreatment and adversity. Finally, it is important to point out that the result of this study does not suggest that all patients seeking care for burnout/stress-related exhaustion are expected to have a history of emotional neglect. However, it may be relevant to consider this aspect for patients struggling with long-term problems that are not being resolved by the current support provided.
